# A first national survey of knowledge, attitudes and behaviours towards schizophrenia, bipolar disorders and autism in France

**DOI:** 10.1186/1471-244X-12-128

**Published:** 2012-08-28

**Authors:** Isabelle Durand-Zaleski, Jan Scott, Frédéric Rouillon, Marion Leboyer

**Affiliations:** 1 Department of Public Health & Psychiatry, AP-HP, URCEco & Henri Mondor-Albert Chenevier Hospitals, 51 avenue du maréchal de Lattre de Tassigny, Creteil, F-94000, France; 2 Faculty of Medicine, University Paris-East, EA 4393 & UMR-S 955, 8 avenue du Général Sarrail, Creteil, F-94000, France; 3 FondaMental Foundation, Fondation de Coopération Scientifique Hôpital, A. Chenevier, 40, Rue de Mesly, Creteil, F-94000, France; 4 Academic Psychiatry, Institute of Neuroscience, Newcastle University, Newcastle upon Tyne, NE1 7RU, UK; 5 CMME –Hopital Sainte Anne, University Paris Descartes, INSERM U 1017 1 Rue Cabanis, F 75014, Paris, France; 6 Psychiatry Genetic, INSERM, U 955, IMRB, Creteil, F-94000, France

**Keywords:** Mental health, Bipolar disorders, Schizophrenia, Autism, Survey, Stigma, Discrimination, Attitudes, Behaviours

## Abstract

**Background:**

In order to support evidence-based policies for reduction of stigma, a better understanding of its components: ignorance (knowledge), prejudice (attitude) and discrimination (behaviour) is necessary. This study explores public perceptions and quantifies stigma for three chronic mental disorders: autism, schizophrenia and bipolar disorders in France.

**Methods:**

Survey of 1000 adults selected from an established market research panel. The 21-item questionnaire explored knowledge, attitudes and behaviours toward each disorder.

**Results:**

Although 95% respondents recognized the names of each disorder fewer than 70% could report specific characteristics and only 33% considered that publically available information was adequate; most respondents identified the media as their main resource. Labeling of conditions in a negative way was frequent (61%) when referring to mental disorders in general, but fell significantly (18%) when linked to an individual with a disorder. Individuals with schizophrenia are assumed to be dangerous; 65% respondents would engage in social distancing from such an individual, versus 29% for bipolar disorders and 7% for autism (p < 0.001). In contrast to other disorders, discrimination against schizophrenia was only partly attenuated in those with familiarity with mental disorders (through personal or family illness).

**Conclusion:**

This first population-based survey in France shows that attitudes towards bipolar disorders and autism are less prejudicial than towards schizophrenia. However, most public attitudes and behaviours towards different disorders appear to be based on assumptions rather than knowledge or evidence suggesting a generic information or anti-stigma programme is unlikely to be effective.

## Background

Stigmatization of individuals with mental disorders has been noted throughout history. However, contemporary research suggests that prejudice and discrimination can change as a function of general shifts in social attitudes or specific alterations in mental health literacy about a disorder and its perceived treatability [1-6]. The varying impact of these attitudes or understanding over time may partly explain why studies of public knowledge of and/or attitudes toward mental disorders produce conflicting findings. For example, since the 1950s, stigma associated with disorders such as depression and anorexia nervosa appears to have declined, partly because people are more likely to attribute the causes to stressful life circumstances, with which they identify more readily [7,8]. Furthermore, familiarity with these disorders (either through personal experience or exposure to the illness experiences of family members or others) has helped reduce social distancing, which in turn decreases the risk of discriminatory behaviours [9,10].

In contrast, stigma associated with psychotic disorders such as schizophrenia may be increasing [3,5,6,9,10]. The reasons are not certain, but it is suggested that unfavourable stereotypes of individuals with schizophrenia (depicting their behaviour as unpredictable and violent) have become more rather than less prevalent. This public image is associated with fearfulness in the community, leading to social avoidance and/or negative discrimination [11]. It is also hypothesized that there have been unexpected negative consequences of promoting ‘biogenic’ aetiological models [12]. Although greater awareness of the causal role of genetic or biological factors has decreased attributions about personal responsibility for developing a severe mental disorder, it appears that biogenic models are more liable to be construed as meaning the prognosis is poor and treatment less likely to improve outcomes [13]. However, these data refer to adults and there is not data regarding views of genetic risk factors in childhood and whether this would worsen or improve attitudes towards such individuals [2].

A number of theoretical frameworks have been developed that explore the above themes, their inter-relationships and any mediating or moderating factors. According to Thornicroft et al. [14], ‘stigma’ is an overarching term that contains three key elements: problems of knowledge (ignorance); problems of attitudes (prejudice); and problems of behaviour (discrimination). This conceptual approach overlaps with social cognition theories of stigma, such as Weiner’s Attribution Model [15,16]: a model describing how cultural stereotypes, attributions about controllability of and responsibility for illness onset, and mediating emotional responses (such as anger or pity), predict the likelihood of helpful or discriminatory behaviours towards individuals with mental disorders. The majority of studies utilizing these theories conceptualize ‘mental disorders’ as a single entity or only focus on schizophrenia [2]. A smaller number of studies compare public knowledge, attitudes and/or reactions toward two disorders (usually depression with schizophrenia), and still fewer contrast depression and schizophrenia with other diagnostic groups such as substance misuse disorders or physical disabilities [10]. European (Germany, Italy and the UK), North American (USA and Canada), Australasian (Australia and New Zealand) and cross-national (Japanese- Australian) studies demonstrate that there are shared stereotypes of psychosis or depression, with many cultural similarities (but also some differences) in attitudes and attributions, emotional responses and tolerance (e.g. [17]). In general, more anger and overt discrimination is directed at individuals with disorders such as substance misuse, purportedly driven by widely held beliefs that the individuals are ‘blameworthy’ and their behaviours are ‘irresponsible’ e.g. [18].

Despite the above research, there are still significant gaps in our understanding of stigma and its consequences. For example, few studies specifically examine mental illnesses with symptoms that span the spectrum of common and severe mental disorders. Bipolar disorders (manic-depression) are the obvious example of such an illness, as the individual may present with depression, mania and/or psychotic symptoms at different time points. However, bipolar disorders are rarely targeted in public stigma research and even fewer studies have differentiated between perceptions of mania and depression (an exception is [16]). Another limitation of current research is that little is known regarding differences in knowledge and attitudes toward childhood compared with adult mental disorders. Studies of stigmatization of children and adolescents have invariably been conducted in isolation from those of adults, or the research has investigated the experiences of the parents of children with neuro-developmental disorders [19,20]. It is unclear whether members of the public are consistent in their attitudes, emotional responses and behaviours toward disorders such as autism that commences during childhood, compared with schizophrenia, depression and mania that usually commences in early adulthood.

Our objectives were to:

a) establish the feasibility of using the internet to recruit a representative sample of adults as a vehicle to undertake a brief survey of knowledge, attitudes and beliefs

b) undertake the first French study of knowledge, attitudes and behaviour towards individuals with mental disorders

c) compare public perceptions of different sub-types of mental disorder, namely a specific childhood developmental disorder (autism), a severe mental disorder (schizophrenia) and a disorder that emulates the presentation of both common and severe mental disorders (bipolar disorders).

## Methods

The study was a collaborative project between a multidisciplinary academic team and an independent contractor with experience of market research (Ipsos Public Affairs). The survey was conducted by Ipsos in accordance with the French laws on privacy. The academic team advised on questionnaire content, had access to all the data and takes full responsibility for the integrity of the analyses and reported research findings. The contractor designed the internet survey instrument, undertook recruitment, performed data collection and was responsible for quality assurance.

### Sample

Individuals aged > =18 years, drawn from an established market research panel available to Ipsos, were contacted via email and invited to complete an online survey between May 8–12, 2009. In the absence of response to the initial contact, individuals were contacted on one further occasion three days later. Recruitment continued until 1000 responses were obtained. To try to ensure the recruited sample was representative of the general adult population of France, sampling was stratified initially for place of residence (taking into account population density) then according to gender, age (in 5 years groupings) and socio-economic status (according to occupation of the head of household).

### Survey questionnaire

In order to ensure this was a brief, user friendly, internet questionnaire that was acceptable to a target population the survey instrument was limited to 21 items written in French. The final set of questions selected targeted key domains such as mental health knowledge (n = 8), attitudes (n = 5) and behaviours (n = 4) towards those with mental disorders and familiarity with mental disorders (n = 4). These items were designed to capture data on key themes examined in previously published questionnaires exploring knowledge, attitudes and beliefs [1-3,9,11,18,21-23]. However, the researchers did not use established questionnaires as (a) using a set of assessment tools for each issue we wished to target would extend the duration of participation to 30–50 minutes, which would lead to significant loss of participants on the internet and was also counter to the idea of a very brief questionnaire, (b) we especially wanted to explore views of disorders spanning childhood and adulthood and different types of presentations and none of the questionnaires or assessment tools available covered this range, (c) we wished to explore whether individuals understand the ‘terminology’ of mental disorders and what actually constitutes the disorder named eg schizophrenia (ie do people know not just the words or names used to describe mental disorders such as schizophrenia, but can they actually spontaneously describe the symptoms and problems that are integral to the disorder with that name), as such we designed some new questions to look at this, (d) we decided that to make this a simple user friendly survey the information on names and symptoms of disorders would be used as an alternative for case vignettes.

Knowledge items (n = 8) included the following- views of the likely prevalence of mental disorders in the general population, causal attributions (eg views of risk factors such as genetic vulnerability, external stressors, etc.), beliefs about controllability (by the individual themselves or via different treatments), beliefs about stability and predictability. Attitudes (n = 5) and behavior (n = 4) were explored by questions that assessed terminology used to describe mental disorders, and reactions such as avoidance or social distancing. Familiarity questions (n = 4) explored issues such as personal or family experiences of mental disorders and predictions about future vulnerability (factors that may act as modifiers of reactions); these were supplemented by questions that explored views on likely help-seeking and probability of self-disclosure.

The item format included ‘yes/no/don’t know’ questions, rank ordering of statements or Likert scale ratings. Respondents were also asked to endorse adjectives, verbs or expressions (from a list provided) that describe responses to mental disorders in general and then individuals with mental disorders specifically. The responses were then further classified qualitatively (eg ‘anger’, ‘pity’, ‘fear’, etc.) to allow the identification of key themes such as sympathetic, empathic or prejudicial [24]. Likewise some questions about predicted behaviours (eg would the respondent be prepared to work alongside someone with a bipolar disorder, schizophrenia or autism) assessed differences in reaction to, or degree of discrimination towards each disorder.

### Statistical analysis

Data was analyzed using the SAS 9.1 statistical software package (SAS Corporation, Cary NC). Statistical significance was set *a priori* at the p < 0.05. For ease of interpretation, all data are presented as percentages unless stated otherwise. Basic statistics, such as chi-squared and rank order testing, were used to explore any specific differences regarding level of knowledge about risk factors for and attitudes towards each of the three disorders studied and when differences were found we then also examined whether there was an age group or gender effect. Missing values were dealt with by excluding a case with missing values from a specific analysis of that variable.

## Results

### Feasibility of conducting a brief internet survey representative of the French population

The target sample of 1000 respondents was recruited within the five day time frame. The sample was 52% female (see Table 1) and the mean age was 45 years (SD 14), about 30% were aged <35 and a similar proportion were aged 55–64 years. About 20% had received tertiary education and the mean monthly household income was 2,300€. About 18% lived in the Paris region, 11% in the south west, with the rest of the sample distributed evenly across the North West, north east and south east. The sample demographics are therefore similar to the adult population of France. Nine hundred and 16 of the 1000 questionnaires were fully completed or provided sufficient responses to allow some or all the questions completed by the individual to be included in the analyses.

**Table 1:** Sociodemographic characteristics of sample

**VARIABLE**		**NUMBER(Total = 1000)**
**Gender**	Male	481
	Female	519
**Age in years**	18–24	109
	25–34	183
	35–44	188
	45–54	181
	55–64	293
	> = 65	46
**Level of Education**	None	21
	Primary	310
	Secondary	412
	Tertiary	257
**Monthly Income**	= < 2000 €	275
	2000 to 3000 €	391
	3000 to 4500 €	209
	Over 4500 €	76
	Not reported	155
**Region of Residence**	North east	242
	North west	238
	South east	231
	South west	112
	Paris & environs	177

### Mental health knowledge

Less than one in five respondents (17%) correctly identified the estimated prevalence of mental disorders to be 21-30%. However, there was almost universal recognition of the names of most mental disorders including the three specific disorders investigated, with 100% respondents recognizing the term autism and 97% and 96% respectively recognizing the terms schizophrenia and bipolar disorders. However, when respondents were asked if they could describe some of the characteristics of these disorders, the proportions decreased to 67% for autism, 53% for schizophrenia and 43% for bipolar disorders. Both awareness and knowledge of the disorders tended to increase with age (up to 55 years) and socioeconomic status but findings were inconsistent across disorders. When age and social status were controlled for, women were more frequently aware than men of mental disorders in general (p < 0.01) and the specific characteristics (p < 0.05).

As shown in Table 2, views about disorders showed significant differences (X^2^ = 55.4; df = 8; p < 0.002): views about the need for treatment (less treatment) and prognosis (better outcome) favoured bipolar disorders; schizophrenia was regarded as similar to autism in having an early onset but viewed as dissimilar in ‘not being diagnosed early’. When offered a list of possible risk factors for each disorders, about two thirds of the sample rated drug or alcohol misuse as the most important risk factor for schizophrenia, 44% rated genetic factors as most important for autism, whilst 67% endorsed emotional stress as the most important risk factor for bipolar disorders; the rank ordering of risk factors for each disorders was statistically significant (p < 0.001).

**Table 2:** Respondents views about course of mental disorders and risk factors for schizophrenia, bipolar disorders and autism*

**Views about course:**	**Schizophrenia**	**Bipolar disorders**	**Autism**	Differences between disorders*
Develops early in life	44%	31%	45%	p < 0.002
Early diagnosis is possible	38%	36%	84%	
Requires lifelong treatment	74%	53%	61%	
With treatment, a person can live a normal life	49%	56%	25%	
It will become more severe / worsen over time	42%	37%	21%	
**Views about risk factors:**				
Drugs & Alcohol Misuse	58%	54%	3%	p < 0.001
Stressful Life Events	52%	67%	23%	
Life Style/Environment	42%	64%	11%	
Parent-Children Interactions	32%	49%	22%	
Genetic Factors	27%	25%	44%	
Did Not Know	17%	12%	21%	

*see text for details: views about course of disorder show % giving a ‘yes’ response; views about risk factors show % ranking each item first or equal first.

Only 33% respondents considered information available about mental disorders and their treatments to be adequate, but they rated the media as a more frequent source of sufficient information (30%) ahead of doctors (27%) or health professionals (21%) or other public services (13%). When asked to rate the effectiveness of a list of different treatments approaches, a minority (<1 in 5) of interviewees expected medication or psychotherapies to be ‘very effective’, whilst >30% considered all treatments to be ineffective or did not know if they were likely to be beneficial. When controlling for age group, women were significantly more likely than men to believe psychological treatments would be very effective (70% v 58%; p < 0.01) and also more likely to endorse medications (74% v 66%).

### Attitudes & behaviours

When asked to provide descriptors of mental disorders, 61% respondents reported pejorative labels (e.g. 47% used the words mad or lunatic). Compassionate descriptors, (e.g. sad or sorrowful), were used by 29% respondents. In comparison, when respondents were asked to provide descriptors of individuals with a mental disorder rather than of mental disorders *per se*, respondents were more likely to use compassionate (34%) and less likely to use negative labels (18%). There was a four-fold likelihood of respondents being compassionate rather than pejorative if considering individuals rather than disorders (Odds Ratio 3.97, 95% Confidence Intervals 1.92 to 8.18; X^2^ = 14.6, df = 1, p < 0.001)

**Figure 1:**
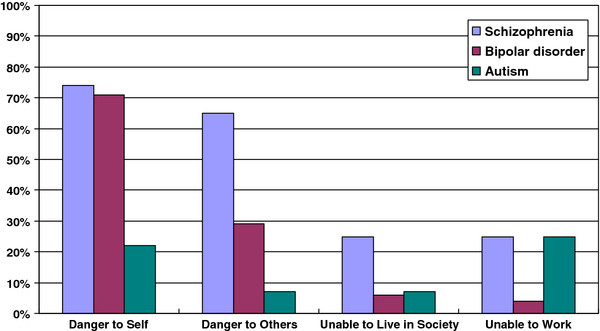
Respondents endorsement of levels of dangerous and social disabilities of individuals with schizophrenia, bipolar disorders and autism.

As shown in Figure 1, respondents viewed individuals with autism or bipolar disorders to be able to live in society, and viewed individuals with bipolar disorders as most likely to be able to work- views that were less likely to be endorsed for schizophrenia. Familiarity with mental disorders was associated with a non-significant trend for greater endorsement of the ability of individuals with autism or bipolar disorders to live in society, but no increase in rate of endorsement for individuals with schizophrenia.

Of the three disorders studied, schizophrenia generated the greatest fear, distrust and desire for social distancing: 65% respondents considered individuals with schizophrenia to be dangerous to others (as shown in Figure 1, this compares with 29% for bipolar disorders and 7% for autism; X^2^ = 12.1, df = 2, p < 0.001). Furthermore, 30% would refuse to work with an individual with schizophrenia (bipolar disorders = 12%, autism = 6%; X^2^ = 11.8, df = 2, p < 0.002), 31% would not want their children in the same class at school (bipolar disorders = 15%, autism = 3%; X^2^ = 21.5, df = 2, p < 0.0002) and 24% would not agree to live with a relative with schizophrenia (bipolar disorders = 11%, autism = 6%; X^2^ = 11.3, df2, p < 0.003). Figure 1 shows that respondents rated 22% of individuals with autism as a danger to themselves, compared with 74% of individuals with schizophrenia and 71% with bipolar disorders (X^2^ = 10.3; df = 2; p < 0.004).

### Familiarity

Although 40% of respondents reported familiarity with mental disorders only 5% (n = 55) reported a previous or current personal history of one or more mental disorder; an additional 34% did report they knew a friend or relative with a mental disorder. Of those with a personal history, 80% (n = 44/55) reported depression or mixed depression with anxiety, 9% (n = 5/55) bipolar disorders and 1.8% (n = 1) schizophrenia. A majority of respondents (65%) considered that they were potentially at risk of developing a mental disorder and two thirds also endorsed the statement that this was also true for members of their family. Interestingly, whilst it was unsurprising that 61% thought the most likely disorder to be experienced in the future would be depression, bipolar disorders (19%) was ranked second, ahead of anxiety (17%) or any other disorder. However, if problems were to occur, 30% of respondents stated that they would not mention it to their relatives, 60% would not mention it to friends, and 95% would not mention it at their workplace.

## Discussion

These data represent the first population-based survey of current public awareness, knowledge and attitudes towards mental illness in France.

### Main findings of this survey

In a sample of 1,000 French persons, name recognition for mental disorders was high and marginally greater than reported in Australia (61%; [12]) and Scotland (72%; [22]). However, awareness of mental disorders (by name) was not necessarily accompanied by knowledge about the characteristics of the specific disorders and there were also limited expectations, especially in men, for treatment efficacy. Negative views of schizophrenia in this sample seem to be similar to those reported in other studies many decades earlier (eg [16]). Interestingly, prejudice, which was evident from the frequent use of negative labels about mental disorders in general, was significantly less overt when descriptors were related to an individual with a mental disorder. This is a relevant finding when considering how we might attempt to reduce stigma, but this simple question represents a novel method of examining this issue.

There were interesting and previously unreported differences in public views of risk factors for each disorder studied, with genetic factors rated as most important in autism but psychosocial factors predominating in bipolar disorders and substance misuse more often rated as a risk factor for schizophrenia. This finding is especially interesting given the fact that there is increasing concern that messages regarding the biogenic nature of mental disorders may have increased rather than decreased negative views of adult mental disorders- this effect was not consistently observed in our study as it appeared to differ across disorders, possibly suggesting that age of individuals with a disorder may influence levels of stigma [3,5,6].

The acknowledged personal experience of mental disorders was lower than predicted and this, plus the fact respondents also stated they would not readily share information with friends or work colleagues (and only 1 in 3 stated they would admit such problems to their family), suggests potentially high levels of self-stigma and fears about negative judgments or rejection by other people. Intriguingly, individuals ranked bipolar disorders as the second most likely future mental health problem (after depression), ahead of other common mental disorders, perhaps indicating that in the public’s mind bipolar disorders are more like depression rather than being severe mental disorders with many similarities with psychoses.

### Specific questions on bipolar disorders, autism and schizophrenia

The responses to questions focusing on bipolar disorders, autism and schizophrenia showed a marked contrast between views of schizophrenia compared with the other disorders. Individuals with schizophrenia were considered dangerous by two out of three respondents and responses indicted that participants continue to have negative view of this disorder with low expectation of patient functioning and a desire for social distance for themselves and their children. Such attitudes were less likely to be expressed regarding bipolar disorders and remarkably rare in response to autism. The fact that negative stereotypes of schizophrenia prevail (despite research that challenges these notions e.g. [25]), may indicate that media views of schizophrenia are still a more powerful influence. Direct support for this hypothesis comes from our finding that this sample acknowledged the media as their main source of information regarding mental disorders. Portrayal by the media of persons with psychosis as dangerous or helpless seem to be the norm while images of such people at school, work or enjoying themselves in the community are rare or absent [26,27]. Historically, there has been less media coverage of autism and bipolar disorders; not only have these disorders only recently begun to attract more public attention, but so far, the images presented in the media have tended to be positive rather than negative and are more often focused on an individual experiences (eg films such as ‘Rain Man’ or reports of celebrities with bipolar disorders) or agreement that there is a need to provide resources to support individuals (eg special school support for autism).

### Study limitations

The company undertaking the majority of opinion surveys in France (Ipsos Public Affairs) provided a sample of sufficient size to ensure only a 3% margin of error. However, the survey was conducted online using individuals already recruited to a panel for market surveys, so by definition respondents were computer literate and accessed the internet regularly. Rates of declared personal history of mental health problems were also lower than expected. This sample may therefore be more homogeneous than in other community studies which may explain why we failed to find more consistent socio-demographic trends as reported in some previous studies [2,23]. The main study limitation is however that in order to make this a brief, internet based questionnaire we have used the concepts and ideas prevalent in the literature but did not use a set of previously validated reliable questionnaires, also some concepts needed to be translated into French (which in some cases may change the subtlety of the original meaning eg whether a person is described as being ‘able to work’ as opposed to ‘unable to work). Hence there may be issues in the nature of the questionnaire that will reduce our ability to compare some of the findings with other studies. Also we used diagnostic labels in some instances instead of case vignettes which will affect the views expressed or items endorsed [13].

## Conclusions

Except for a short media campaign on Depression in 2007 (3 minutes of information broadcasted during prime time on a national network), France has not undertaken an anti-stigma campaign such as “Changing in Minds” in UK (1998–2002),”Beyond Blue” in Australia (2001–2005), “Like Minds Like Mine “ in New Zealand (1997–2004) , the 'See Me' –national campaign in Scotland ( 2002–2004) or BASTA (Bavarian Anti-Stigma-Action). In eight countries where such public awareness campaigns took place, the programs contributed to a modest improvement in public knowledge of and attitude toward mental disorders [28]. The current survey demonstrates that if the French public is to benefit from a similar venture, any campaign should take into account the fact that (a) as attitudes towards individuals with a disorder are more benign than those expressed about mental disorders in general, it would be beneficial to build on approaches that specifically use personal testimonies and raise the visibility of individuals with mental disorders who are living normal lives, as this is likely to be more effective than generic campaigns trying to de-stigmatize the disorders [29] and (b) the public appear to differentiate between autism (individuals were not viewed as being personally responsible, nor where they seen as dangerous), bipolar disorders (viewed differently from severe disorders and rated as likely to have a good outcome even in the absence of treatment) and schizophrenia (viewed by the majority as likely to have a poor outcome, potentially dangerous and cannot live independently or work in the community). The latter suggest strongly that a campaign that treated mental disorders as a single entity is unlikely to be successful. Furthermore, any strategy that does not involve re-educating the media or fails to gain their support in disseminating a more balanced view is likely to fail. It may be that a stepped model would be a more helpful strategy, starting by building upon knowledge and more benign attitudes regarding depression, introducing information about bipolar disorders, then gradually making links between the more severe aspects of this disorder and psychoses in general, with a view to identifying similarities between schizophrenia and highlighting policy initiatives such as early intervention programmes that have improved clinical and social outcomes. Such programmes cannot rely on ‘information’ alone as it is known that whilst this is important, it is insufficient on its own to change attitudes and behaviours. So additional strategies that raise the visibility of individuals with a disorder, rather than just of the disorder will be important, especially projects that use an interactive approach to engaging people in dialogue [30,31]. This survey however confirms that stigma and prejudice towards schizophrenia is equally prevalent in France as elsewhere. However, it also demonstrates that public awareness, knowledge, attitudes and behaviours towards mental disorders varies between different presentations. Future initiatives to challenge stigma and discrimination should therefore consider whether disorder-specific initiatives will be more effective than general approaches to mental illnesses.

## Authors’ contributions

IDZ has acted as a speaker, consultant or investigator and received honoraria from the following firms: Johnson &Johnson (Janssen-Cilag), Novartis, MSD, Pasteur, Sanofi-Aventis, GSK, Astra-Zeneca, Medtronic. JLS has acted as a speaker for, attended advisory boards or had unrestricted educational grants from Astra-Zeneca, BMS-Otsuka, Eli-Lilly, GSK, Janssen-Cilag, Lundbeck, Sanofi-Aventis and Servier. ML has acted as a speaker for Servier and Astra-Zeneca. All authors read and approved the final manuscript.

## Pre-publication history

The pre-publication history for this paper can be accessed here:

http://www.biomedcentral.com/1471-244X/12/128/prepub
